# Surface ECG–based complexity parameters for predicting outcomes of catheter ablation for nonparoxysmal atrial fibrillation: efficacy of fibrillatory wave amplitude

**DOI:** 10.1097/MD.0000000000029949

**Published:** 2022-08-05

**Authors:** Jong-Il Park, Seung-Woo Park, Min-Ji Kwon, Jeon Lee, Hong-Ju Kim, Chan-Hee Lee, Dong-Gu Shin

**Affiliations:** a Yeungnam University College of Medicine, Daegu, Korea; b Division of Cardiology, Department of Internal Medicine, Yeungnam University Medical Center, Daegu, Korea; c Lyda Hill Department of Bioinformatics, University of Texas Southwestern Medical Center, Dallas, TX, USA.

**Keywords:** nonparoxysmal atrial fibrillation, radiofrequency catheter ablation, 12-lead ECG, complexity analysis

## Abstract

Catheter ablation (CA) is a well-established therapy for rhythm control in atrial fibrillation (AF). However, CA outcomes for persistent AF remain unsatisfactory because of the high recurrence rate despite time-consuming efforts and the latest ablation technology. Therefore, the selection of good responders to CA is necessary. Surface electrocardiography (sECG)-based complexity parameters were tested for the predictive ability of procedural termination failure during CA and late recurrence of atrial arrhythmias (AA) after CA. A total of 130 patients with nonparoxysmal AF who underwent CA for the first time were investigated. A 10-second sECG of 4 leads (leads I, II, V_1_, and V_6_) was analyzed to compute the fibrillatory wave amplitude (FWA), dominant frequency (DF), spectral entropy (SE), organization index (OI), and sample entropy (SampEn). The study endpoints were procedural termination failure during CA and late (≥1 year) AA recurrence after CA. In the multivariate analysis, FWA in lead V_1_ and DF in lead I were independent predictors of successful AF termination during CA (*P* <.05). The optimal cut-off values for FWA in lead V_1_ and DF in lead I were 60.38 μV (area under the curve [AUC], 0.672; *P* = .001) and 5.7 Hz (AUC, 0.630; *P* = .016), respectively. The combination of FWA of lead V_1_ and DF of lead I had a more powerful odds ratio for predicting procedural termination failure (OR, 8.542; 95% CI, 2.938–28.834; *P* < .001). FWA in lead V_1_ was the only independent predictor of late recurrence after CA. The cut-off value is 65.73 μV which was 0.634 of the AUC (*P* = .009).

These sECG parameters, FWA in lead V_1_ and DF in lead I, predicted AF termination by CA in patients with nonparoxysmal AF. In particular, FWA in lead V_1_ was an independent predictor of late recurrence of AA after CA.

## 1. Introduction

Although radiofrequency catheter ablation (CA) is a well-established therapy used to achieve rhythm control in atrial fibrillation (AF), its outcomes for persistent AF, especially long-standing AF, are unsatisfactory despite the use of up-to-date ablation technology.^[1,2]^ This is because recurrence rates remain high and serious complications can still occur despite time-consuming efforts.^[3,4]^ Therefore, it is important to identify good responders to CA to ensure better outcomes and avoid unnecessary procedural risks.

Numerous predictors based on AF type, clinical risk factors, imaging, circulating biomarkers, genetic predictors, and electrocardiographic and electrophysiological parameters have been studied to aid in the identification of patients with AF for a high probability of ablation success.^[5,6]^ Several scoring systems based purely on clinical parameters have been developed to predict AF recurrence after CA. However, these multivariable scoring systems remain modestly accurate and lack successful implementation in clinical settings.^[7]^

Surface electrocardiography (sECG), an easily accessible, noninvasive test used to detect AF, provides key information regarding the integrity and electrophysiological properties of the atrial myocardium.^[8,9]^ Accordingly, fibrillatory waves on sECG are surrogate markers that represent the atrial electrical and structural status related to AF remodeling.

Metrics for characterizing fibrillatory electrical activity have focused on quantifying the degree of organization using AF cycle length,^[10–13]^ dominant frequency (DF),^[14,15]^ f-wave amplitude (FWA),^[14,16,17]^ organization index (OI), and related spectral features.^[14,18]^ Although some of these parameters have been reported to predict arrhythmia recurrence after AF ablation, a single parameter was evaluated in most studies and the results were inconsistent. Therefore, this study aimed to compare the efficacy of various sECG-based complexity parameters in predicting both procedural termination failure and long-term recurrence of nonparoxysmal AF.

## 2. Materials and Methods

### 2.1. Study patients

**Figure 1.**
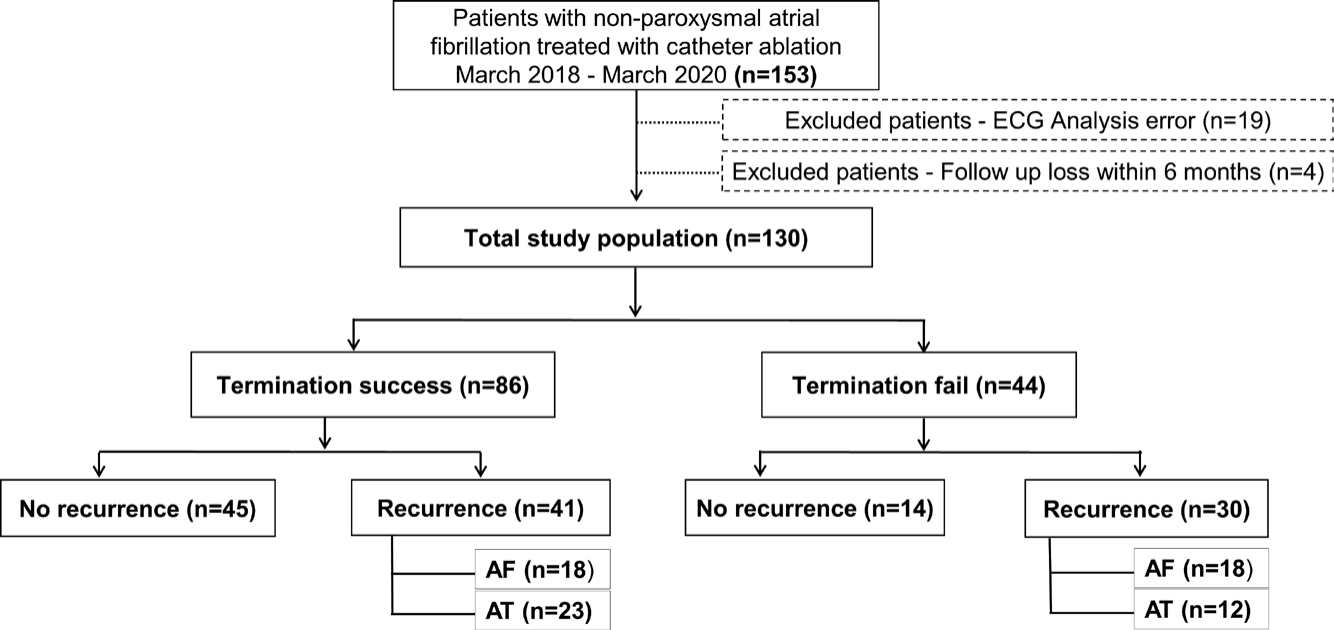
Study population selection process. AF = atrial fibrillation, AT = atrial tachycardia, ECG = electrocardiogram.

This study enrolled 153 consecutive patients with nonparoxysmal drug-refractory AF who underwent their first CA between March 2018 and March 2020. After excluding 19 patients with corrupted ECG signals and 4 lost to follow-up, 130 patients were ultimately analyzed (Fig. 1). The study protocol was approved by the institutional review board of Yeungnam University Hospital (IRB No:-2021-04-022). The requirement for informed consent was waived because of the retrospective nature of the study. The study complied with the principles of the Declaration of Helsinki.

### 2.2. sECG analysis and data processing

All the patients had AF when they entered the electrophysiology laboratory. Each 12-lead sECG was collected using an EP Workmate system (EP WorkMate™ System, Abbott, St. Paul, MN, USA) at a sampling rate of 2 kHz for at least 60 seconds before the CA. A 60-second 12-lead ECG recording was exported for later analysis, from which a 10- second ECG epoch was selected for the primary analysis. Among the 12 leads of the conventional ECG, the tracings of 4 lead sets (leads I, II, V_1_, and V_6_), which are known to reflect localized right and left atrial activity and show optimal prediction performances, were selected for ECG analysis.^[19–21]^ To extract an atrial activity signal from each of the leads, an event synchronous adaptive filter (ESAF)-based method previously developed by the authors was used.^[22]^ In detail, a wavelet filter, of which passing bandwidth corresponds to approximately 1–32Hz, was applied to reject motion artifacts and power-line interference. Then, R peaks were detected from the filtered lead signal and those were converted into an impulse train signal, of which impulses are synchronized with the R peaks. The filtered lead and the impulse train signals were fed to the ESAF as a primary input and a reference signal, respectively. Finally, as an output of the ESAF, a ventricular activity canceled atrial activity signal was obtained. This atrial activity signal was used for further analysis. The DF (expressed in Hertz),^[23]^ OI,^[24]^ spectral entropy (SE)^[24]^ for the frequency domain analysis, sample entropy (SampEn),^[25]^, and FWA^[11]^ were computed for the time-domain analysis. The computational formula for each parameter has been described elsewhere.^[23–26]^ All computations were performed using custom-made software (developed by J Lee) in MATLAB (Mathworks Inc., Natick, MA, USA).

### 2.3. CA protocol

Our routine approach for AF ablation has been previously described.^[26]^ Briefly, left atrial (LA) geometry was acquired using a spiral mapping catheter (IBI Inquiry Optima, St. Jude Medical, Inc., St. Paul, MN, USA) in combination with the EnSite NavX™ system (Endocardial Solutions). A TactiCath™ contact force ablation catheter (St. Jude Medical, Inc.) was used in all cases. Wide circumferential pulmonary vein isolation (PVI) across the ipsilateral pulmonary veins was performed, and PVI was confirmed by the loss of pulmonary vein potentials (entry block) and failure to capture the LA during pacing from all bipoles of the circular ring catheter (output: 10 mA; pulse width: 2 ms; exit block). Power-controlled RF energy was delivered at 25–35 W for 20–40 seconds for each lesion. Lower power (20–25 W) and duration settings were used for ablation of the posterior LA wall close to the esophagus. After PVI, if AF persisted, then a 3D-electroanatomical LA complex fragmented atrial electrogram (CFAE) map was constructed using an Advisor™ HD Grid mapping catheter (Abbott). CFAE was ablated in the LA until complete elimination of the fractionated atrial electrograms.

A CFAE was identified when the mean fractionation interval was <120 ms in the following settings: 6-second-long acquisition with a 50-ms refractory period (width, 10 ms; sensitivity, 0.5–1.0 mV).^[27]^ After CFAE ablation, if patients remained in AF, the sinus rhythm was restored by cardioversion.

### 2.4. Study endpoints

The study endpoints were AF termination during ablation and recurrence 1 year after ablation in patients with nonparoxysmal AF. AF termination was defined as the conversion of AF to atrial tachycardia, atrial flutter, or sinus rhythm. An attempt was made to map and ablate all mappable atrial tachyarrhythmias when the AF was organized into atrial tachycardia or atrial flutter. Recurrence at 1 year was defined as symptomatic or asymptomatic atrial arrhythmia (AF, atrial flutter, or atrial tachycardia) lasting > 30 seconds documented on sECG, Holter monitor, or event recorder after 3 months of ablation procedure without antiarrhythmic use.

### 2.5 Postablation management and follow-up

Outpatient visits were made 1 week after discharge and then every 1 or 2 months thereafter. Standard sECG was performed at each visit, and the patient’s symptoms were checked. Holter monitoring was performed when symptoms were present; otherwise, the subjects underwent Holter monitoring every three months from the end of the blanking period.

### 2.6. Echocardiographic examination

All echocardiographic measurements were performed according to the American Society of Echocardiography recommendations. The left ventricular ejection fraction (LVEF) was examined using Simpson’s method. We measured LA dimensions as the anteroposterior diameter in the parasternal long-axis view. We measured the LA volume by the prolate ellipse method using apical 4-chamber and parasternal long-axis views at ventricular end-systole.^[28]^ The LA volume index was calculated as the LA volume corrected by body surface area. Right ventricular systolic pressure (RVSP) was measured as the sum of the trans-tricuspid gradient and estimated right atrial pressure. The trans-tricuspid gradient was estimated using the modified Bernoulli equation: *P* = 4 × *V*^2^, where *V* is the peak tricuspid regurgitation velocity in m/s. The right atrial pressure was estimated using the caval respiratory index.^[29]^

### 2.7 Statistical methods

Data are expressed as a number (%) for categorical variables and as mean ± standard deviation or median and interquartile range (25th–75th) for continuous variables. We checked the distribution normality of continuous variables using the Kolmogorov–Smirnov test. If data normality was proven, these variables were analyzed using parametric Student’s *t*-test. Nonnormally distributed data were examined using the nonparametric Mann–Whitney *U*-test. Categorical data were compared using the chi-squared test or Fisher’s exact test. Next, a logistic regression model was applied for variables with *P* values <.05 in the univariate analysis, and the odds ratio (OR) was calculated to find independent predictors of success during and after the procedure. For each sECG parameter that showed a significant intergroup difference, receiver operating characteristic curves were computed to obtain cut-off values for optimal sensitivity and specificity. The area under the curve (AUC) was calculated using a prediction performance index. Statistical analyses were performed using IBM SPSS version 22.0 (IBM Corp., Armonk, NY, USA). Statistical significance was set at *P* < .05.

## 3. Results

### 3.1. Patients’ characteristics

The mean age was 60 ± 10 years (female, 16.9%). Patients who had prior ablation, prior cardiac surgery, valvular heart diseases or implantable cardiac devices and were older than 80 years were excluded. The median AF duration was 23 (5–64) months. The mean follow-up duration was 16 ± 8 months. The CHADS2VASc score was 2.36 ± 1.29. Long-standing persistent AF was observed in 80 patients (62%). The clinical characteristics of all study patients and differences in the characteristics according to end-points are summarized in Table 1. The demographic variables, AF duration, frequency of long-standing AF and comorbid diseases were similar between the groups, regardless of whether AF was terminated during the procedure. The patients in whom AF was terminated during the procedure were more likely to have a smaller LA AP diameter (41.5 ± 5.1 mm vs. 46.5 ± 4.9 mm), LA volume (59.9 ± 17.8 mL vs. 80.2 ± 24.8 mL), LAVI (33.2 ± 10.0 vs. 43.0 ± 12.7), LA CT volume (139.0 ± 34.4 mL vs. 173.2 ± 44.7 mL) than those of not terminated patients (*P* < .001 for all). No differences in the demographic variables, AF duration, frequency of long-standing AF and comorbid diseases, and LA size were found between groups according to late recurrence.

**Table 1:** Clinical characteristics of the study patients, and difference of characteristics according to the end-points.

Variable	Overall population (n = 130)	Procedural termination (n = 130)			Late recurrence (n = 130)		
		Yes (n = 86)	No (n = 44)	*P* value	No (n = 59)	Yes (n = 71)	*P* value
Clinical parameter							
Age (y)	59.7 ± 9.6	60.8 ± 10.0	57.6 ± 9.3	.081	60.6 ± 10.9	59.0 ± 8.5	.356
Female	22 (16.9)	18 (13.8)	4 (3.1)	.137	11 (18.6)	11 (15.5)	.403
BMI (kg/m^2^)	26.1 ± 3.4	25.8 ± 3.1	26.7 ± 3.7	.151	26.3 ± 3.6	25.8 ± 3.1	.324
Long standing persistent	80 (61.5)	54 (41.5)	26 (20.0)	.411	33 (55.9)	47 (66.2)	.155
AF duration (mo)	23.0 (5.0–64.0)	23.0 (6.5–61.0)	20.0 (4.0–72.0)	.890	16.0 (5–51.5)	25.5 (5.5–67.5)	.390
Hypertension	70 (50.8)	42 (32.3)	28 (21.5)	.138	35 (59.3)	35 (49.3)	.167
Diabetes	26 (20.0)	17 (13.1)	9 (6.9)	.549	14 (23.7)	12 (16.9)	.227
Congestive heart failure	55 (42.3)	35 (26.9)	20 (15.4)	.369	25 (42.4)	30 (42.3)	.565
Vascular disease	29 (22.3)	21 (16.2)	8 (6.2)	.282	16 (27.1)	13 (18.3)	.161
Stroke	30 (23.1)	17 (13.1)	13 (10.0)	.151	11 (18.6)	19 (26.8)	.189
CHADS2VASc score	2.36 ± 1.29	2.40 ± 1.37	2.30 ± 1.13	.660	2.54 ± 1.39	2.21 ± 1.19	.147
Echocardiographic parameter							
LVEF	57.6 ± 8.5	57.6 ± 8.4	57.6 ± 8.8	.964	57.2 ± 8.8	58.0 ± 8.3	.588
LA AP diameter (mm)	43.2 ± 5.6	41.5 ± 5.1	46.5 ± 4.9	<.001	42.9 ± 5.3	43.5 ± 5.8	.532
LA volume (mL)	62.7 ± 22.5	59.9 ± 17.8	80.2 ± 24.8	<.001	65.4 ± 21.0	67.9 ± 23.8	.523
LAVI (mL/m^2^)	36.5 ± 11.9	33.2 ± 10.0	43.0 ± 12.7	<.001	36.0 ± 10.9	37.0 ± 12.7	.617
RVSP (mmHg)	25.7 ± 10.5	28.6 ± 6.7	31.3 ± 8.8	.063	25.5 ± 12.0	25.8 ± 9.3	.875
3D CT findings							
LA CT volume (mL)	150.7 ± 41.4	139.0 ± 34.4	173.2 ± 44.7	<.001	148.8 ± 39.4	152.2 ± 43.2	.641
Catheter ablation strategy							
CFAE	93 (71.5)	52 (60.5)	41 (93.2)	<.001	39 (66.1)	54 (76.1)	.244

### 3.2 sECG-based complexity parameters

The sECG parameters according to the study end-points are summarized in Table 2. In patients in whom AF was terminated during the procedure, the FWA in lead V_1_ was significantly larger (75.47 ± 26.18 vs. 64.76 ± 29.79, *P* = .038), and the DF of lead I (5.549 ± 1.011 vs. 6.036 ± 1.057, *P* = .012) and lead II (5.505 ± 0.918 vs. 5.886 ± 0.992, *P* = .031) were significantly smaller than those of patients in whom AF was not terminated. The SE of lead II was lower (3.009 ± 0.579 vs. 3.277 ± 0.482, *P* = .006) in patients in whom AF was terminated. As a predictor of late recurrence, the FWA of lead V_1_ was larger (77.96 ± 27.51 vs. 66.80 ± 27.24, *P* = .023), and the SE of lead V_1_ was smaller (2.625 ± 0.706 vs. 2.925 ± 0.721, *P* = .032) in patients with no recurrence of AF did not recur. No differences were found in other sECG parameters.

**Table 2:** Surface electrocardiography parameters according to the end-points.

Variable	Overall population (n = 130)	Procedural termination (n = 130)			Late recurrence (n = 130)		
		Yes (n = 86)	No (n = 44)	*P* value	No (n = 59)	Yes (n = 71)	*P* value
f wave amplitude (μV)							
Lead I	44.54 ± 16.38	44.93 ± 16.08	44.78 ± 17.10	.706	45.34 ± 16.91	43.87 ± 16.01	.613
Lead II	65.58 ± 22.18	68.28 ± 22.30	60.30 ± 21.21	.052	69.57 ± 24.46	62.26 ± 19.66	.061
Lead V1	71.82 ± 27.82	75.47 ± 26.18	64.76 ± 29.79	.038	77.96 ± 27.51	66.80 ± 27.24	.023
Lead V6	57.34 ± 25.91	56.50 ± 21.38	58.97 ± 33.26	.609	59.42 ± 21.33	55.60 ± 29.21	.405
Dominant frequency (Hz)							
Lead I	5.714 ± 1.049	5.549 ± 1.011	6.036 ± 1.057	.012	5.563 ± 1.057	5.839 ± 1.032	0.135
Lead II	5.634 ± 0.957	5.505 ± 0.918	5.886 ± 0.992	.031	5.471 ± 0.830	5.769 ± 1.038	.077
Lead V1	5.852 ± 1.211	5.793 ± 1.316	5.968 ± 0.998	.602	5.810 ± 1.185	5.969 ± 1.027	.417
Lead V6	5.585 ± 1.264	5.551 ± 1.248	5.650 ± 1.305	.293	5.542 ± 0.970	5.620 ± 1.470	.447
Organization index							
Lead I	0.698 ± 0.109	0.707 ± 0.115	0.679 ± 0.092	.170	0.705 ± 0.118	0.692 ± 0.101	.540
Lead II	0.705 ± 0.089	0.708 ± 0.082	0.698 ± 0.102	.574	0.694 ± 0.076	0.713 ± 0.098	.232
Lead V1	0.752 ± 0.128	0.754 ± 0.141	0.748 ± 0.099	.482	0.733 ± 0.141	0.768 ± 0.116	.257
Lead V6	0.712 ± 0.102	0.718 ± 0.102	0.702 ± 0.101	.388	0.730 ± 0.108	0.699 ± 0.095	.091
Spectral entropy							
Lead I	3.292 ± 0.507	3.281 ± 0.506	3.314 ± 0.514	.724	3.303 ± 0.529	3.267 ± 0.455	.701
Lead II	3.100 ± 0.561	3.009 ± 0.579	3.277 ± 0.482	.006	3.104 ± 0.562	3.091 ± 0.566	.904
Lead V1	2.715 ± 0.721	2.649 ± 0.754	2.844 ± 0.640	.125	2.625 ± 0.706	2.925 ± 0.721	.032
Lead V6	3.277 ± 0.519	3.274 ± 0.556	3.381 ± 0.426	.078	3.274 ± 0.536	3.285 ± 0.486	.910
Sample entropy							
Lead I	0.127 ± 0.016	0.127 ± 0.015	0.129 ± 0.018	.401	0.127 ± 0.015	0.128 ± 0.017	.884
Lead II	0.124 ± 0.013	0.123 ± 0.014	0.125 ± 0.013	.448	0.122 ± 0.013	0.125 ± 0.013	.261
Lead V1	0.125 ± 0.018	0.123 ± 0.019	0.129 ± 0.012	.052	0.122 ± 0.021	0.127 ± 0.013	.201
Lead V6	0.118 ± 0.016	0.119 ± 0.016	0.117 ± 0.015	.582	0.117 ± 0.015	0.119 ± 0.016	.542

Table 3 shows the results of univariate and multivariate analyses for predicting procedural termination failure or late recurrence (bad outcomes). In the univariate analysis of procedural termination failure, LA CT volume, FWA in lead V_1_, DF in lead I, DF in lead II and SE in lead II were statistically significant (all, *P* < .05). In the univariate analysis of late recurrence, the FWA in lead V_1_ (*P* = .027) and SE in lead V_1_ (*P* = .040) were statistically significant. Multivariate analysis revealed that, LA CT volume (OR, 1.029; 95% CI, 1.015–1.043; *P* < .001) was an independent predictor of procedural termination failure during CA. Among the sECG parameters, FWA in lead V_1_ (OR, 0.981; 95% CI, 0.964–0.999; *P* = .034) and DF in lead I (OR, 2.037; 95% CI, 1.297–3.197; *P* = .002) were powerful predictors of procedural termination failure. A low FWA in lead V1 or a high DF in lead I predicted procedural termination failure. For the prediction of late recurrence, FWA in lead V_1_ was the only independent predictor of late recurrence of AF after CA (OR, 0.985; 95% CI, 0.972–0.998; *P* = .027). A low FWA in lead V1 predicted late recurrence of AF after CA.

**Table 3:** Multivariate logistic regression analysis for prediction of procedural termination failure or late recurrence.

Variable	Univariate analysis		Multivariate analysis (Model I)	
	OR (95% CI)	*P* value	OR (95% CI)	*P* value
Procedural termination failure				
LA CT volume(mL)	1.024 (1.012–1.036)	<.001	1.027 (1.014–1.041)	<.001
FWA V_1_ (μV)	0.985 (0.970–0.985)	.042	0.981 (0.964–0.999)	.034
DF I (Hz)	1.582 (1.097–2.281)	.014	2.032 (1.300–3.177)	.002
DF II (Hz)	1.538 (1.035–2.285)	.033	1.357 (0.810–2.274)	.246
SE II	2.532 (1.232–5.205)	.011	2.129 (0.949 –4.778)	.067
Late recurrence				
FWA V_1_ (μV)	0.985 (0.972–0.998)	.027	0.985 (0.972–0.998)	.027
SE V_1_	1.842 (1.027–3.305)	.040	1.041 (0.618–1.753)	.880

### 3.3. Optimal cut-off value for predicting procedural termination failure and recurrence after CA

**Figure 2.**
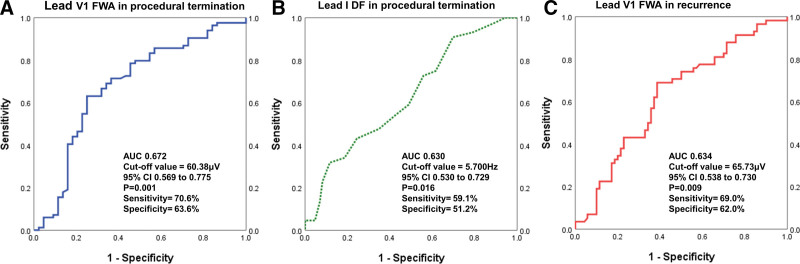
Receiver operating characteristic curve of FWA in lead V_1_ and DF in lead I according to the outcomes of CA. ROC curve of lead V_1_ FWA (A) and lead I DF (B) for procedural termination failure, and lead V_1_ FWA for late recurrence (C). The area under the curve (AUC), cut-off value, *P*-value, 95% confidence interval (CI), sensitivity, and specificity of each parameter are shown. CA = catheter ablation, DF = dominant frequency, FWA = fibrillatory wave amplitude.

Figure 2 shows the receiver operating characteristic (ROC) curves for the optimal cut-off values that predict procedural termination failure and late recurrence after CA (bad outcomes). The AUC for predicting AF termination during CA was 0.672 for the FWA in lead V_1_ and 0.630 for the DF in lead I. When the cut-off value of the FWA in lead V_1_ was 60.38 μV, the sensitivity and specificity were 70.6% and 63.6%, respectively (95% CI, 0.569–0.775, *P* = .001). When the cut-off value of the DF in lead I was 5.70 Hz, the sensitivity and specificity were 59.1% and 51.2%, respectively (95% CI, 0.530–0.729, *P* = .016). The AUC for predicting late AF recurrence of FWA in lead V_1_ was 0.634 and the cut-off value was 65.73 μV. Sensitivity and specificity were 69.0% and 62.0%, respectively.

### 3.4. *Combination of FWA in lead V*_*1*_ *and DF in lead I had higher predictive value for AF termination during CA*

Table 4 shows that the combination of a lower FWA in lead V_1_ (< 60.38 μV) and a higher DF in lead I (> 5.7 Hz) had a more powerful odds ratio for predicting procedural termination failure (OR, 8.542; 95% CI, 2.938–24.834; *P* < .001) than a lower FWA in lead V_1_ (< 60.38 μV; OR, 4.919; 95% CI, 2.020–11.983; *P* < .001) or a higher DF in lead I (> 5.7 Hz; OR, 3.099; 95% CI, 1.217–7.890; *P* = .018) alone.

**Table 4:** Multivariate analysis of the cut-off value depending on the surface electrocardiography parameters in procedural termination failure.

Variable	Multivariate analysis (Model II)		Multivariate analysis (Model III)	
	OR (95% CI)	*P* value	OR (95% CI)	*P* value
Procedural termination failure				
LA CT volume(mL)	1.029 (1.015–1.044)	<.001	1.030 (1.016–1.044)	<.001
Low FWA V_1_ (< 60.38 μV)	4.919 (2.020–11.983)	<.001		
High DF I (> 5.700 Hz)	3.099 (1.217–7.890)	.018		
Combination of low FWA V_1_ and high DF I			8.542 (2.938–24.834)	<.001

## 4. Discussion

The main findings of the present study were that sECG-based complexity parameters are useful for predicting procedural termination failure of AF and late recurrence of atrial arrhythmias (bad outcomes) after CA for nonparoxysmal AF. In particular, FWA in lead V_1_ is a powerful independent predictor of both procedural termination failure and late recurrence. DF in lead I was also an independent parameter for predicting procedural termination failure during CA. The combination of the FWA of lead V_1_ (< 60.38 μV) and the DF of lead I (> 5.70 Hz) was 8.54 times more likely to predict failure of AF termination during CA. In the present study, LA CT volume was the only parameter, except for sECG, that predicted procedural termination failure during CA. Previous studies have shown a statistically significant relationship between CA success and LA size.^[30,31]^

### 4.1. sECG-based complexity parameters as predictors of CA outcome

The clinical risk prediction of AF recurrence in patients who have undergone CA is limited.^[7]^ The standard 12-lead ECG is an attractive method for the assessment of AF complexity level because it is an easily accessible, noninvasive test. Recently, many complexity parameters derived from the 12-lead ECG, such as the AF cycle length,^[10,12,13]^ FWA^[11,14,16,17,32]^ DF^[14,15,23,33,34]^ OI,^[14,24,32]^ SE,^[14,24]^, and SampEn^[14,25,32]^ have been proposed. Although several parameters showed encouraging results in predicting treatment outcomes, the results were not consistent.^[32]^ A single parameter has been tested in most previous studies. In the present study, we adopted five parameters (FWA and SampEn for the time-domain analysis, and DF, OI, and SE for the frequency domain analysis of fibrillatory waves) and compared them. Several studies have demonstrated that the FWA is a predictor of procedural termination failure and AF recurrence.^[11,14,16,17]^ Maximal amplitude of ≥ 0.07 mV in V_1_/lead II predicted AF termination by ablation and patients with FWA < 0.05 mV in lead V_1_ had higher AF recurrence.^[11]^ This cutoff value is similar to the FWA cutoff value (< 60.38 μV) proposed.^[11,16]^ We showed that the FWA of lead V_1_ was a powerful independent predictor of both procedural termination failure and late recurrence. Procedural termination failure during CA is a prognostically important endpoint of CA for nonparoxysmal AF.^[10,35,36]^

Regarding the predictive efficacy of DF, patients with a high DF in lead aVL (DF ≥ 6.9 Hz) and V_1_ DF of ≥ 7.1 Hz showed a lower success rate of persistent AF ablation.^[33]^ In another study, the best electrographic predictor of AF termination was DFs in the left atrial appendage (DF < 6.5 Hz) and lead II (DF < 5.9 Hz).^[34]^ Likewise, the DF of lead I (< 5.7 Hz) was an independent parameter for predicting procedural termination failure during CA in our study. Taken together, patients with a lower FWA of lead V_1_ (< 60.38 μV) and/or higher DF of lead I (> 5.7 Hz) would have a lower AF termination rate and higher late recurrence rate. FWA was the best predictor of the outcome.

### 4.2. Optimal sECG lead for prediction of CA outcome

The lead that showed the best predictive value was different depending on the study, in which it was lead I in one study,^[16]^ lead aVF and V_6_ in another study,^[14]^ or lead V_1_^[11,17]^ as in our study. The role of lead V_1_ FWA in predicting CA outcomes is challenging to determine. Fibrillatory waves of sECG lead V_1_ closely reflect the right atrium and, to a lesser degree, the left atrial activity.^[37]^ On surface ECG, the amplitude of fibrillatory waves is dependent on the magnitude of the underlying voltage, which is related to the magnitude of the remaining viable atrial muscle.^[17]^ Patients with permanent AF had a greater extent of fibrosis than those with paroxysmal AF.^[38]^ The structural remodeling of atrial fibrosis theoretically leads to a decrease in muscle fiber activation, which affects the FWA voltage.^[39,40]^ Therefore, lower FWA in lead V_1_ implies a reduction in right atrial voltage with increased low voltage areas as a result of advanced structural remodeling with progression of AF.

The efficacy of sECG-based parameters in predicting treatment outcomes was not consistent. DF, OI, SampEn, and FWA were not able to predict arrhythmia recurrence following ablation in a previous study.^[32]^ This might be due to the different ablation techniques, strategies, and compositions of the study patients. In this study, SE, SampEn, and OI showed no ability to predict procedural termination failure or arrhythmia recurrence after CA.

### 4.3. Clinical implications

To date, the selection of patients with nonparoxysmal AF who benefited from CA has been at the physician’s discretion. As a noninvasive tool, sECG is being used by physicians to easily treat and evaluate AF patients in real-world practice. These sECG-based complexity parameters from the results of this study can identify patients who are likely to benefit from CA. Among these, FWA was the most powerful sECG-derived predictor. This study proposes the quantitative cut-off value of FWA in lead V_1_ (procedural termination failure: 60.38 μV, late recurrence: 65.73 μV) and DF in lead I (procedural termination failure: 5.70 Hz) for selecting patients who benefited from CA by predicting procedural termination failure. This can help physicians decide whether to recommend CA to patients with nonparoxysmal AF. AF complexity parameters quantified from sECG can be employed in a clinical setting to predict treatment outcomes and ultimately guide AF management.

The incorporation of artificial intelligence and machine learning technology into AF management in the future would facilitate the selection of nonparoxysmal AF patients who benefit most from CA, the assessment of procedure-related risk, specific ablation target selection, and prediction of ablation outcomes in AF. Overall, this would lead to an improvement in patient-tailored CA procedures for treating nonparoxysmal AF.

### 4.4. Limitations

In this study, CFAE ablation was used as adjunctive ablation after PVI. CA outcomes, such as AF termination or long-term recurrence, will vary depending on which technique among CFAE ablation or additional linear lesions as adjunctive ablation strategy after PVI is used. The outcomes would also differ depending on electrophysiology laboratory staff experience and proficiency. Since the cut-off values of FWA and DF are determined by the ablation success rate, the cutoff values may vary. For these reasons, this is a major limitation of this study. Second, owing to the small sample size of a single center and relatively short follow-up period, our findings should be validated in a large prospective multicenter cohort study prior to their application in clinical practice. Third, we selected only four leads (I, II, V_1_, and V_6_) among the 12 ECG leads for analysis; therefore, we might have missed the optimal lead with better predictability. Simultaneous analysis of the FWA in several ECG leads may improve CA long-term outcome prediction in persistent AF compared with predictors based on a single lead.^[16]^ The combination of clinical parameters and/or the best-predicting ECG parameters will further improve the prediction performance.

## 5. Conclusion

Noninvasive sECG-based complexity parameters, especially FWA in lead V_1_ and DF in lead I, are effective for prediction of the procedural termination failure of AF by a single-time CA and long-term recurrence of atrial arrhythmias in patients with nonparoxysmal AF.

## Author contributions

Conceptualization: JIP, CHL, DGS.

Data curation: JIP, SWP, MJK, HJK.

Formal analysis: JIP, SWP, MJK.

Software: JL.

Supervision: DGS.

Visualization: JIP, DGS.

Writing - original draft: JIP, SWP, MJK, DGS.

Writing – review & editing: JIP, DGS.

## References

[1] Tilz RR Rillig A Thum AM. Catheter ablation of long-standing persistent atrial fibrillation: 5-year outcomes of the Hamburg Sequential Ablation Strategy. J Am Coll Cardiol. 2012;60:1921–9. 23062545 10.1016/j.jacc.2012.04.060

[2] Brooks AG Stiles MK Laborderie J. Outcomes of long-standing persistent atrial fibrillation ablation: a systematic review. Heart Rhythm. 2010;7:835–46. 20206320 10.1016/j.hrthm.2010.01.017

[3] Steinbeck G Sinner MF Lutz M. Incidence of complications related to catheter ablation of atrial fibrillation and atrial flutter: a nationwide in-hospital analysis of administrative data for Germany in 2014. Eur Heart J. 2018;39:4020–9. 30085086 10.1093/eurheartj/ehy452 PMC6269631

[4] Gupta A Perera T Ganesan A. Complications of catheter ablation of atrial fibrillation: a systematic review. Circ Arrhythm Electrophysiol. 2013;6:1082–8. 24243785 10.1161/CIRCEP.113.000768

[5] Vlachos K Letsas KP Korantzopoulos P. Prediction of atrial fibrillation development and progression: current perspectives. World J Cardiol. 2016;8:267–76. 27022458 10.4330/wjc.v8.i3.267 PMC4807315

[6] Weng LC Preis SR Hulme OL. Genetic predisposition, clinical risk factor burden, and lifetime risk of atrial fibrillation. Circulation. 2018;137:1027–38. 29129827 10.1161/CIRCULATIONAHA.117.031431 PMC5840011

[7] Dretzke J Chuchu N Agarwal R. Predicting recurrent atrial fibrillation after catheter ablation: a systematic review of prognostic models. Europace. 2020;22:748–60. 32227238 10.1093/europace/euaa041 PMC7203634

[8] Peter RH Morris JJ Jr. McIntosh HD. Relationship of fibrillatory waves and P waves in the electrocardiogram. Circulation. 1966;33:599–606. 5937556 10.1161/01.cir.33.4.599

[9] Holm M Pehrson S Ingemansson M. Non-invasive assessment of the atrial cycle length during atrial fibrillation in man: introducing, validating and illustrating a new ECG method. Cardiovasc Res. 1998;38:69–81. 9683908 10.1016/s0008-6363(97)00289-7

[10] Rostock T Salukhe TV Steven D. Long-term single- and multiple-procedure outcome and predictors of success after catheter ablation for persistent atrial fibrillation. Heart Rhythm. 2011;8:1391–7. 21699825 10.1016/j.hrthm.2011.04.012

[11] Nault I Lellouche N Matsuo S. Clinical value of fibrillatory wave amplitude on surface ECG in patients with persistent atrial fibrillation. J Interv Card Electrophysiol. 2009;26:11–9. 19404588 10.1007/s10840-009-9398-3

[12] Fiala M Bulkova V Sknouril L. Sinus rhythm restoration and arrhythmia noninducibility are major predictors of arrhythmia-free outcome after ablation for long-standing persistent atrial fibrillation: a prospective study. Heart Rhythm. 2015;12:687–98. 25576779 10.1016/j.hrthm.2015.01.004

[13] Kochhauser S Jiang CY Betts TR. Impact of acute atrial fibrillation termination and prolongation of atrial fibrillation cycle length on the outcome of ablation of persistent atrial fibrillation: a substudy of the STAR AF II trial. Heart Rhythm. 2017;14:476–83. 28011328 10.1016/j.hrthm.2016.12.033

[14] Lankveld T Zeemering S Scherr D. Atrial fibrillation complexity parameters derived from surface ECGs predict procedural outcome and long-term follow-up of stepwise catheter ablation for atrial fibrillation. Circ Arrhythm Electrophysiol. 2016;9:e003354. 26823480 10.1161/CIRCEP.115.003354

[15] Hidalgo-Munoz AR Tome AM Latcu DG. Empirical mode decomposition of multiple ECG leads for catheter ablation long-term outcome prediction in persistent atrial fibrillation. Annu Int Conf IEEE Eng Med Biol Soc. 2015;2015:105–8. 26736211 10.1109/EMBC.2015.7318311

[16] Zarzoso V Latcu DG Hidalgo-Munoz AR. Non-invasive prediction of catheter ablation outcome in persistent atrial fibrillation by fibrillatory wave amplitude computation in multiple electrocardiogram leads. Arch Cardiovasc Dis. 2016;109:679–88. 27402153 10.1016/j.acvd.2016.03.002

[17] Cheng Z Deng H Cheng K. The amplitude of fibrillatory waves on leads aVF and V1 predicting the recurrence of persistent atrial fibrillation patients who underwent catheter ablation. Ann Noninvasive Electrocardiol. 2013;18:352–8. 23879275 10.1111/anec.12041 PMC6932451

[18] Alcaraz R Hornero F Rieta JJ. Electrocardiographic spectral features for long-term outcome prognosis of atrial fibrillation catheter ablation. Ann Biomed Eng. 2016;44:3307–18. 27221509 10.1007/s10439-016-1641-3

[19] Dibs SR Ng J Arora R. Spatiotemporal characterization of atrial activation in persistent human atrial fibrillation: multisite electrogram analysis and surface electrocardiographic correlations--a pilot study. Heart Rhythm. 2008;5:686–93. 18452870 10.1016/j.hrthm.2008.01.027

[20] Guillem MS Climent AM Millet J. Noninvasive localization of maximal frequency sites of atrial fibrillation by body surface potential mapping. Circ Arrhythm Electrophysiol. 2013;6:294–301. 23443619 10.1161/CIRCEP.112.000167 PMC4292880

[21] Meo M Zarzoso V Meste O. Spatial variability of the 12-lead surface ECG as a tool for noninvasive prediction of catheter ablation outcome in persistent atrial fibrillation. IEEE Trans Biomed Eng. 2013;60:20–7. 23033326 10.1109/TBME.2012.2220639

[22] Lee J Song MH Shin DG. Event synchronous adaptive filter based atrial activity estimation in single-lead atrial fibrillation electrocardiograms. Med Biol Eng Comput. 2012;50:801–11. 22718318 10.1007/s11517-012-0931-7

[23] Chiarugi F Varanini M Cantini F. Noninvasive ECG as a tool for predicting termination of paroxysmal atrial fibrillation. IEEE Trans Biomed Eng. 2007;54:1399–406. 17694860 10.1109/TBME.2007.890741

[24] Uldry L Van Zaen J Prudat Y. Measures of spatiotemporal organization differentiate persistent from long-standing atrial fibrillation. Europace. 2012;14:1125–31. 22308083 10.1093/europace/eur436

[25] Alcaraz R Abasolo D Hornero R. Optimal parameters study for sample entropy-based atrial fibrillation organization analysis. Comput Methods Programs Biomed. 2010;99:124–32. 20392514 10.1016/j.cmpb.2010.02.009

[26] Lee CH Lee SH Park KW. The impact of the CHA2DS2-VASc score on recurrence of atrial fibrillation after a single catheter ablation and atrial remodeling in patients with non-valvular atrial fibrillation. Int J Arrhythm. 2017.

[27] Calo L De Ruvo E Sciarra L. Diagnostic accuracy of a new software for complex fractionated electrograms identification in patients with persistent and permanent atrial fibrillation. J Cardiovasc Electrophysiol. 2008;19:1024–30. 18554211 10.1111/j.1540-8167.2008.01219.x

[28] Jiamsripong P Honda T Reuss CS. Three methods for evaluation of left atrial volume. Eur J Echocardiogr. 2007;9:351–5. 17658300 10.1016/j.euje.2007.05.004

[29] Kircher BJ Himelman RB Schiller NB. Noninvasive estimation of right atrial pressure from the inspiratory collapse of the inferior vena cava. Am J Cardiol. 1990;66:493–6. 2386120 10.1016/0002-9149(90)90711-9

[30] Beukema WP Elvan A Sie HT. Successful radiofrequency ablation in patients with previous atrial fibrillation results in a significant decrease in left atrial size. Circulation. 2005;112:2089–95. 16203925 10.1161/CIRCULATIONAHA.104.484766

[31] Lo LW Tai CT Lin YJ. Predicting factors for atrial fibrillation acute termination during catheter ablation procedures: implications for catheter ablation strategy and long-term outcome. Heart Rhythm. 2009;6:311–8. 19251203 10.1016/j.hrthm.2008.11.013

[32] McCann A Vesin JM Pruvot E. ECG-based indices to characterize persistent atrial fibrillation before and during stepwise catheter ablation. Front Physiol. 2021;12:654053. 33859573 10.3389/fphys.2021.654053 PMC8042333

[33] Murase Y Inden Y Shibata R. The impact of the dominant frequency of body surface electrocardiography in patients with persistent atrial fibrillation. Heart Vessels. 2020;35:967–76. 32016538 10.1007/s00380-020-01563-7

[34] Uetake S Miyauchi Y Osaka M. Frequency analysis of surface electrocardiograms (ECGs) in patients with persistent atrial fibrillation: correlation with the intracardiac ECGs and implications for radiofrequency catheter ablation. J Arrhythmia. 2014;30:453–9.

[35] O’Neill MD Wright M Knecht S. Long-term follow-up of persistent atrial fibrillation ablation using termination as a procedural endpoint. Eur Heart J. 2009;30:1105–12. 19270341 10.1093/eurheartj/ehp063

[36] Elayi CS Di Biase L Barrett C. Atrial fibrillation termination as a procedural endpoint during ablation in long-standing persistent atrial fibrillation. Heart Rhythm. 2010;7:1216–23. 20206323 10.1016/j.hrthm.2010.01.038

[37] Husser D Stridh M Cannom DS. Validation and clinical application of time-frequency analysis of atrial fibrillation electrocardiograms. J Cardiovasc Electrophysiol. 2007;18:41–6. 17229299 10.1111/j.1540-8167.2006.00683.x

[38] Platonov PG Mitrofanova LB Orshanskaya V. Structural abnormalities in atrial walls are associated with presence and persistency of atrial fibrillation but not with age. J Am Coll Cardiol. 2011;58:2225–32. 22078429 10.1016/j.jacc.2011.05.061

[39] Quah JX Dharmaprani D Tiver K. Atrial fibrosis and substrate based characterization in atrial fibrillation: time to move forwards. J Cardiovasc Electrophysiol. 2021;32:1147–60. 33682258 10.1111/jce.14987

[40] Ballesteros G Ravassa S Bragard J. Association of left atrium voltage amplitude and distribution with the risk of atrial fibrillation recurrence and evolution after pulmonary vein isolation: an ultrahigh-density mapping study. J Cardiovasc Electrophysiol. 2019;30:1231–40. 31077505 10.1111/jce.13972

